# Active Versus Passive Infrared Thermography for Skin Cancer Detection: A Diagnostic Accuracy Study

**DOI:** 10.3390/cancers18050829

**Published:** 2026-03-04

**Authors:** Fernando Malheiros, Evelyn Rocha Silva, Pedro Noronha Fagundes, Jose Jeronimo Rabelo Faria, Raquel Descie Veraldi Leite, Isabela Guerra, Vanessa d Andretta Tanaka, Bruno Augusto Alvares, Vinicius de Lima Vazquez

**Affiliations:** 1 Unnawave LTDA, Rua Padre Rodolfo, 300, Vila Ema, São José dos Campos 12243-080, SP, Brazil; fernandomalheiros@gmail.com (F.M.); evnrochasilva@gmail.com (E.R.S.); pedronfagundes@gmail.com (P.N.F.); jeronimo.rfaria@gmail.com (J.J.R.F.); 2 Educational & Research Institute, Barretos Cancer Hospital, Fundação Pio XII, Rua Antenor Duarte Villela, 1331, Dr. Paulo Prata, Barretos 14784-400, SP, Brazil; isabelaguerra90@gmail.com (I.G.); vanessatanaka.derma@gmail.com (V.d.A.T.); brunoalvares_91@hotmail.com (B.A.A.); viniciusvazquez@gmail.com (V.d.L.V.)

**Keywords:** infrared thermography, skin cancer, diagnostic accuracy, thermal imaging, temperature differential

## Abstract

Background: Infrared thermography has emerged as a non-invasive imaging modality capable of capturing physiological alterations associated with skin cancer; however, its diagnostic utility remains insufficiently characterized. This study aimed to compare the diagnostic performance of active (cooling-induced) and passive infrared thermography in distinguishing benign from malignant skin lesions. Methods: In this clinical study conducted at Barretos Cancer Hospital, 64 individuals contributed 100 skin lesions, of which 68 (56 malignant, 12 benign) met criteria for diagnostic accuracy analysis. Long-wave infrared images were acquired under steady-state conditions (passive thermography) and after standardized cooling (active thermography). Diagnostic performance was assessed using thermal contrast patterns and temperature differentials (Δ*T*), with histopathology as the reference standard. Results: Active thermography markedly outperformed passive imaging. Active thermography achieved a sensitivity of 91.1% and specificity of 75.0%, whereas passive thermography demonstrated low sensitivity (17.9%) despite perfect specificity (100%). Malignant lesions displayed substantially higher Δ*T* values, particularly during active thermal recovery. ROC analysis confirmed the superiority of the active technique (AUC = 0.871) over passive imaging (AUC = 0.650), with an optimal Δ*T* cutoff of 0.70 °C yielding high discriminative accuracy. Conclusions: Active infrared thermography enhances thermal contrast and provides significantly improved diagnostic sensitivity compared with passive imaging. As a low-cost, non-invasive technique, active thermography shows promise as a complementary tool for skin cancer triage, particularly in settings with limited access to dermatologic evaluation.

## Simple Summary

Skin cancer is highly curable when detected early, but access to specialist evaluation and advanced diagnostic tools remains limited in many settings. Infrared thermography is a non-invasive imaging technique that measures skin temperature and may help identify abnormal tissue. In this study, we compared two thermographic approaches—passive imaging at rest and active imaging after brief cooling—to evaluate their ability to distinguish benign from malignant skin lesions. We found that active thermography was substantially more sensitive in detecting malignant lesions, while passive imaging missed most cancers despite high specificity. Temperature differences between lesions and surrounding skin were more pronounced after cooling, improving diagnostic discrimination. These findings suggest that active infrared thermography may serve as a low-cost, non-invasive complementary tool to support early skin cancer detection, particularly in screening or triage settings where access to dermatologic care is limited.

## 1. Introduction

Skin cancer is the most common malignancy worldwide and represents approximately one-third of all cancers diagnosed in Brazil [1,2,3]. The principal subtypes include basal cell carcinoma (BCC), squamous cell carcinoma (SCC), and cutaneous melanoma, each strongly associated with cumulative ultraviolet (UV) exposure [1,4]. Although melanoma accounts for only 4–6% of cases, it remains the most aggressive form due to its high metastatic potential [1,2,3]. Importantly, early detection enables cure rates exceeding 90%, emphasizing the need for accurate and accessible diagnostic strategies [1,2,5].

Initial assessment of suspicious lesions relies mainly on clinical examination and dermoscopy, the latter significantly improving diagnostic accuracy but remaining dependent on clinician expertise and subjective interpretation [6,7,8]. Misclassification may lead to delayed diagnosis or unnecessary excisions [4,9], highlighting the need for complementary, objective tools to support clinical decision-making.

Infrared thermography has gained renewed interest as a non-invasive imaging modality capable of detecting thermal abnormalities associated with neoplastic processes. Skin lesions may present distinct temperature patterns arising from altered vascularization, metabolic activity, or inflammation [10,11]. Two approaches are commonly employed: passive thermography, which captures spontaneous thermal emission at equilibrium, and active (dynamic) thermography, which applies controlled thermal stimulation, such as cooling, to enhance contrast and reveal perfusion-related differences between lesions and surrounding tissue [12,13,14,15]. By amplifying thermal gradients, active thermography may improve the detection of subtle malignant changes not apparent under steady-state conditions.

Although several studies have explored the use of infrared thermography in dermatology and in the detection of skin cancer [10,11,13,14,15,16,17], the literature remains disparate, characterized by unstandardized methodologies, variable diagnostic thresholds, and heterogeneous lesion types. Such variability limits comparability across studies and hinders clinical translation. Furthermore, few investigations have systematically quantified diagnostic accuracy metrics—including sensitivity, specificity, and overall accuracy—using histopathology as the reference standard.

In this context, the present clinical study aimed to evaluate and compare the diagnostic performance of passive and active infrared thermography in distinguishing benign from malignant skin lesions. By analyzing temperature differentials (Δ*T*) and applying standardized classification criteria against biopsy results, we sought to determine the accuracy, sensitivity, and specificity of each technique and to clarify their potential role as complementary tools in the early detection of skin cancer.

## 2. Materials and Methods

### 2.1. Study Design and Setting

This open-label, non-randomized diagnostic accuracy study was conducted at Barretos Cancer Hospital (Brazil) between 1 August and 20 December 2023 (approximately four months). The study compared the diagnostic performance of passive infrared thermography and active infrared thermography (index tests) in distinguishing malignant from benign skin lesions, using histopathological examination as the reference standard. The study was approved by the BCH ethics committee (CAAE number 72847223.2.0000.5437). All participants provided informed consent.

### 2.2. Participants

Adults (≥18 years) presenting with at least one skin lesion clinically suspected of malignancy and scheduled for surgical excision were eligible. Exclusion criteria included ulcerated lesions, lesions > 10 cm in diameter, atypical examination areas that impeded image acquisition, extensive dermatoses, and autoimmune diseases that could be exacerbated by cold exposure. Participation was voluntary, and written informed consent was obtained prior to data collection. A total of 100 lesions were initially evaluated; however, only 76 lesions with complete thermal and histopathological data and adequate cooling response were included in the diagnostic accuracy analysis. Premalignant lesions were excluded from accuracy calculations. All imaging procedures were performed in a temperature-controlled room maintained at 22 °C (71.60 °F), with no direct airflow over the examination area. Participants underwent a 10 min acclimatization period prior to image acquisition to minimize transient thermal fluctuations related to recent physical activity or environmental exposure. The infrared camera was calibrated at the beginning of each session according to the manufacturer’s specifications.

### 2.3. Thermographic Imaging Procedures (Index Tests)

#### 2.3.1. Passive Thermography

Thermal images were acquired using an Optris Xi400 long-wave infrared camera (Optris GmbH, Berlin, Germany), spectral range 8–14 μm. The device was positioned perpendicular to the lesion at a standardized distance of 35 cm, ensuring consistent field of view and spatial resolution. Emissivity was set to 0.98 to correspond to human skin. Images were obtained under steady-state thermal conditions without external thermal stimulation.

#### 2.3.2. Active Thermography

Immediately after obtaining passive images, a commercial gel pack cooled to 0 °C (verified immediately before application) was applied directly to the lesion and a margin of at least 1–2 cm of surrounding healthy skin for 15 s with gentle and uniform contact. The cooling duration was standardized for all lesions to ensure reproducibility. No excessive pressure was applied in order to avoid mechanical compression effects on local perfusion.

Immediately after removal of the cooling stimulus, infrared image acquisition was resumed without delay to capture the dynamic thermal recovery phase. Sequential thermal images were recorded during the early recovery period, allowing visualization of lesion-to-background temperature differences over time. For analysis, the image demonstrating the maximum temperature differential (Δ*T*) between the lesion and adjacent healthy skin during the recovery phase was selected. This approach aimed to capture peak perfusion-related contrast induced by controlled thermal stress.

### 2.4. Reference Standard (Histopathology)

All lesions were surgically excised according to institutional protocols and evaluated by board-certified pathologists. Histopathological diagnosis served as the gold standard for classification as malignant or benign.

### 2.5. Image Processing and Temperature Differential Analysis

**Figure 1:**
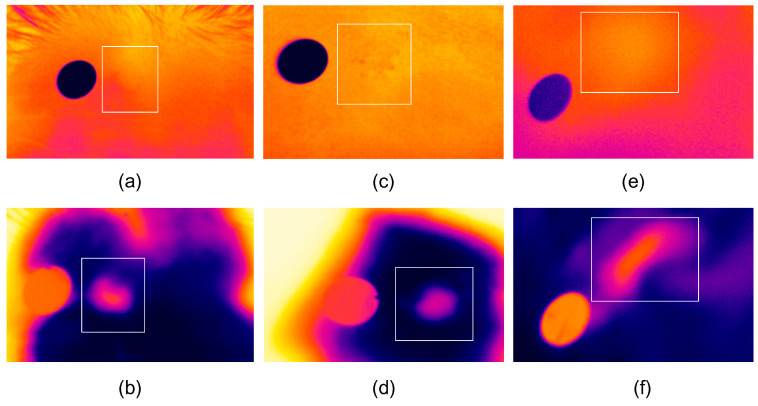
Passive (PT) and active (AT) thermography of the same lesion in representative cases: (**a**) PT in basal cell carcinoma (BCC); (**b**) AT in the same BCC lesion; (**c**) PT in squamous cell carcinoma (SCC); (**d**) AT in the same SCC lesion; (**e**) PT in melanoma; (**f**) AT in the same melanoma lesion. Each PT/AT pair corresponds to the same patient, allowing direct visual comparison of thermal patterns before and after standardized cooling.

A reflective adhesive marker was placed adjacent to each lesion to aid region-of-interest (ROI) identification. Using the camera software, mean temperatures were extracted from the lesion area (*T*_lesion_) and a surrounding area of healthy skin (*T*_healthy_). The temperature differential was calculated as Δ*T* = *T*_lesion_ − *T*_healthy_. The results of passive thermography were compared to those of active thermography, as illustrated in Figure 1.

### 2.6. Diagnostic Classification Rules

Classification criteria were prespecified. A lesion was categorized as thermographically malignant if: (i) it displayed a clearly visible thermal hotspot as assessed independently by two evaluators, or (ii) Δ*T* ≥ 0.5 °C. The Δ*T* ≥ 0.5 °C threshold was defined a priori based on established thermographic principles and was applied for the primary binary classification analysis. Lesions lacking visible thermal contrast and presenting Δ*T* < 0.5 °C were categorized as benign. Two evaluators performed independent readings; although not completely blinded, they were unaware of histopathological results at the time of classification. Two evaluators jointly reviewed the thermographic images and reached a consensus classification. Interobserver agreement was not formally quantified.

Receiver operating characteristic (ROC) analysis was subsequently performed using Δ*T* as a continuous variable to explore optimal data-driven thresholds; these exploratory cutoffs were derived independently from the prespecified 0.5 °C criterion.

Active thermography requires uniform cooling before thermal recovery image acquisition. The inability to achieve this condition resulted in the exclusion of 24 lesions, leaving 76 valid tests for thermal analysis, as originally recorded in the manuscript. After removing the premalignant lesions, the final number of lesions included in the diagnostic accuracy analysis was 68, which corresponds to the dataset used to calculate sensitivity, specificity, and accuracy.

### 2.7. Statistical Analysis

Primary outcomes included sensitivity, specificity, overall accuracy, and balanced accuracy of passive and active thermography. Diagnostic accuracy measures and their 95% confidence intervals were derived using 2 × 2 contingency tables. Given the imbalanced distribution of malignant and benign lesions in this tertiary-care cohort, sensitivity and specificity were prioritized as primary performance measures, as they represent class-conditional probabilities independent of disease prevalence. Balanced accuracy, defined as the mean of sensitivity and specificity, was additionally calculated to mitigate potential inflation of overall accuracy in the presence of class imbalance.

Receiver operating characteristic (ROC) analysis was performed using Δ*T* as a continuous classifier of malignancy. The area under the curve (AUC) was used as a prevalence-independent measure of discriminatory performance. Optimal cutoff values were determined using the Youden index. Differences in paired proportions (passive vs. active thermography) were assessed using McNemar’s test, appropriate for paired diagnostic data.

Continuous variables (Δ*T* values) were summarized as means and standard deviations or medians and interquartile ranges, depending on distribution. Statistical analyses were performed using SPSS Statistics v.27 (IBM Corp., Armonk, NY, USA) and Spyder IDE v5.4.1 (open-source Python development environment).

## 3. Results

A total of 64 individuals were included, contributing 100 skin lesions that underwent thermographic evaluation. After quality control of the thermal acquisition process, 68 lesions (56 malignant and 12 benign) met criteria for inclusion in the diagnostic accuracy analysis.

### 3.1. Sample Characteristics

Table 1 summarizes the demographic and clinical characteristics of the study population. Participants had a mean age of 65.54 ± 14.90 years (range 31–94), and 53.1% were female. Most individuals (75.4%) presented with a single suspicious lesion, whereas the remainder exhibited multiple lesions. The face and scalp were the most frequently affected anatomical regions (48%), followed by the trunk (22%) and upper limbs (20%). Among all 100 lesions initially assessed, 68% were confirmed as malignant, 23% as benign, and 9% as premalignant; the latter were excluded from diagnostic accuracy analyses. Regarding the histopathological distribution of the lesions, basal cell carcinoma was the most prevalent subtype (48%), followed by squamous cell carcinoma (18%) and melanoma (2%). The agreement between the initial clinical suspicion and the final histopathological diagnosis was 85.55%.

**Figure 2:**
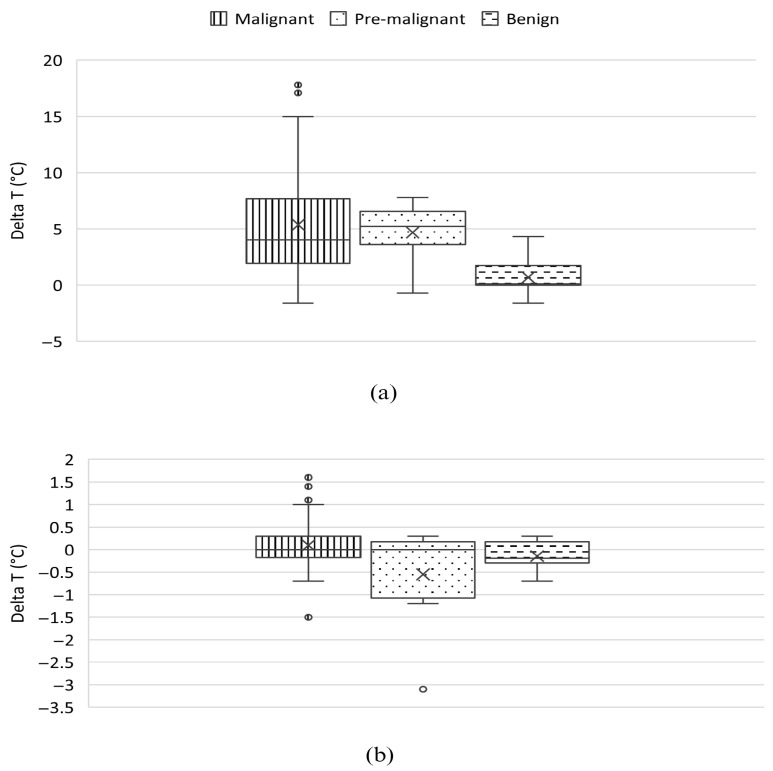
Boxplots showing the distribution of temperature deltas (Δ*T*) for malignant, premalignant, and benign lesions in (**a**) Active Thermography and (**b**) Passive Thermography. The box represents the interquartile range (IQR), the horizontal line within the box indicates the median, and the whiskers extend to the most extreme values within 1.5 × IQR from the first and third quartiles. Circles represent outliers (values beyond 1.5 × IQR), and the “×” symbol denotes the mean value.

Temperature differential values (Δ*T*) were analyzed for both active and passive thermography. Mean Δ*T* values for malignant, premalignant, and benign lesions are presented in Supplementary Table S1, and their distribution is illustrated in Figure 2. Active thermography generated markedly higher Δ*T* values for malignant lesions compared with benign lesions, resulting in greater thermal contrast during the recovery phase. Passive thermography showed Δ*T* values close to zero for most lesions, with limited separation between malignant and benign findings. In active thermography, malignant lesions demonstrated the widest range of Δ*T* values, consistent with the heterogeneous vascular and metabolic profiles expected in neoplastic tissues.

### 3.2. Diagnostic Performance of Active and Passive Thermography

Table 2 presents the 2 × 2 contingency matrices for both thermographic modalities relative to histopathology. Active thermography correctly identified 51 of 56 malignant lesions, while passive thermography identified only 10. Regarding benign lesions, passive thermography showed perfect agreement with histopathology, correctly identifying all 12 lesions as benign. In contrast, active thermography produced three false-positive classifications.

Diagnostic accuracy metrics are summarized in Table 3. Active thermography demonstrated high sensitivity (91.1%; 95% CI, 80.4–97.0) and accuracy (88.2%; 95% CI, 78.1–94.8), with moderate specificity (75.0%; 95% CI, 42.8–94.5). Conversely, passive thermography yielded markedly lower sensitivity (17.9%; 95% CI, 8.9–30.4) while maintaining perfect specificity (100%; 95% CI, 73.5–100). The overall accuracy of the passive method was 32.4% (95% CI, 21.5–44.8). In the paired comparison using McNemar’s test (Supplementary Table S2), active thermography outperformed passive thermography in sensitivity (*p* < 0.0001), whereas the difference in specificity did not reach statistical significance (*p* = 0.25), likely reflecting the limited number of benign lesions.

ROC analysis was performed using Δ*T* as a continuous classifier of malignancy for both active and passive infrared thermography. Active thermography demonstrated excellent discriminative capacity, with an area under the curve (AUC) of 0.871, indicating strong separation between malignant and benign lesions. The optimal cutoff value identified by the Youden index was 0.70 °C, corresponding to a sensitivity of 0.911 and specificity of 0.750. This cutoff was derived from ROC analysis using Δ*T* as a continuous predictor and is distinct from the prespecified Δ*T* ≥ 0.5 °C threshold applied in the primary binary classification. These findings corroborate the high sensitivity observed in the binary accuracy analysis and highlight the enhanced lesion-to-background contrast generated during thermal recovery.

**Figure 3:**
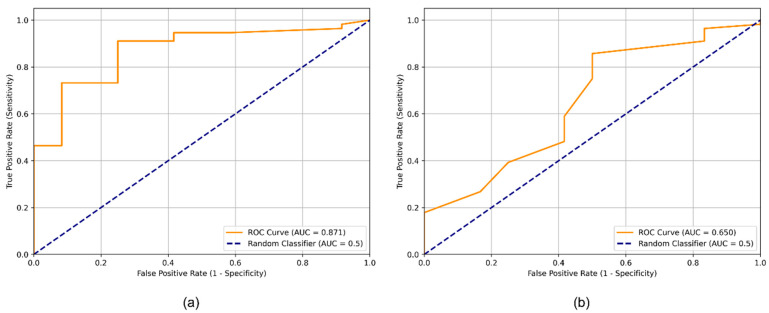
Receiver operating characteristic (ROC) curves for (**a**) active and (**b**) passive infrared thermography using Δ*T* as a continuous predictor of malignancy.

In contrast, passive thermography exhibited modest discriminative performance, with an AUC of 0.650. The optimal cutoff (−0.20 °C) yielded a sensitivity of 0.857 but substantially lower specificity (0.500), reflecting the limited thermal contrast captured at baseline and the overlap between malignant and benign Δ*T* values in the passive phase. The comparative ROC curves for active and passive thermography are shown in Figure 3, demonstrating the superior diagnostic performance of the active approach.

## 4. Discussion

This study evaluated the diagnostic performance of active and passive infrared thermography for differentiating benign from malignant skin lesions and demonstrated that active thermography markedly outperformed passive imaging. These findings are consistent with prior work showing that dynamic or stress-induced thermography enhances physiological contrast by revealing abnormal vascular responses that are not evident under thermal equilibrium [12,14,15]. The superior performance of the active approach observed in this study, particularly its sensitivity of 91.1%, aligns with established mechanistic principles in thermography, whereby cooling-induced thermal recovery amplifies metabolic and angiogenic differences between malignant and non-malignant tissues [11,12,15]. Accordingly, active thermography should be interpreted as a complementary tool rather than a replacement for established diagnostic pathways, including dermoscopy and histopathological examination.

Unlike many previous investigations that evaluated either passive or dynamic thermography in isolation, the present study performed a direct head-to-head comparison of active and passive infrared thermography within the same clinical cohort, using identical acquisition conditions and histopathological diagnosis as the reference standard. By applying both modalities to the same lesions and performing paired statistical comparisons, this design minimizes inter-study variability and allows a more rigorous assessment of the incremental diagnostic value of dynamic thermal stimulation. Furthermore, the use of prespecified classification criteria alongside ROC-based continuous analysis provides both clinically grounded and data-driven perspectives on diagnostic performance, strengthening methodological transparency compared with prior exploratory reports.

Beyond cutaneous oncology, dynamic thermography has demonstrated similar performance gains in other tumor types. Recent investigations in breast cancer have shown that stress-induced thermal imaging combined with computational models enhances early lesion discrimination compared with static thermal acquisition [18,19]. In thyroid malignancy, artificial intelligence–enhanced infrared thermography has also improved diagnostic stratification compared with visual assessment alone [20]. These cross-organ findings support the physiological rationale observed in the present study, reinforcing that dynamic thermal perturbation is a reproducible mechanism for amplifying tumor-related vascular signals.

Passive thermography, by contrast, detected only a small fraction of malignant lesions (17.9%), performing substantially below values reported in earlier exploratory studies in which passive infrared imaging achieved moderate sensitivity for melanoma metastases or advanced tumors [10,13]. This discrepancy likely reflects differences in lesion characteristics. Most cancers in the present cohort were early-stage basal cell carcinomas (BCCs) and squamous cell carcinomas (SCCs), which typically exhibit lower resting thermal contrast than metastatic melanoma or inflamed tumors. Shada et al. [13], for example, evaluated cutaneous melanoma metastases and reported considerably higher passive thermal detectability than observed in this study. Such tumors are generally larger, more vascularized, and more metabolically active, explaining their stronger passive thermal signature.

Importantly, recent feasibility analyses have further demonstrated that passive thermal contrast is particularly vulnerable to environmental noise, camera resolution, and data degradation effects, potentially reducing diagnostic reliability in real-world screening conditions [21]. These findings help contextualize the modest performance of passive thermography observed in our study and highlight the necessity of controlled acquisition protocols when static thermal methods are used.

The Δ*T* findings observed in this study also mirror patterns described in the thermal imaging literature. Malignant lesions demonstrated higher Δ*T* values, particularly following cooling, reflecting the well-documented metabolic and vascular behavior of skin cancers [12,14,15]. The broad Δ*T* range observed among malignant lesions (−1.6 °C to 17.5 °C) is compatible with the known heterogeneity of angiogenesis in non-melanoma skin cancers [4]. Tumor vascularity directly influences thermal appearance, and biological studies have consistently shown that malignant lesions may maintain elevated heat emission due to increased blood flow and disorganized vasculature [10,12,15].

Recent comprehensive reviews integrating bio-heat transfer modeling and quantitative thermal analysis have emphasized that Δ*T* alone may not fully capture lesion complexity unless combined with temporal recovery curves or computational feature extraction [22]. This perspective reinforces the importance of dynamic assessment and supports the ROC-based analysis applied in the present study.

Differences across histopathological subtypes provide further insight. Basal cell carcinomas were less conspicuous thermographically, a finding consistent with morphological studies demonstrating sparse intratumoral vascularity and predominantly peripheral vessel distribution in BCCs [23,24]. In contrast, SCCs exhibited the highest Δ*T* values, corroborating reports of denser vascular networks and increased VEGF expression relative to BCCs [24,25]. These vascular characteristics likely contributed to the stronger thermal contrast observed in SCCs, particularly under dynamic cooling conditions, where perfusion-driven heat recovery becomes more apparent.

Premalignant lesions displayed intermediate thermal behavior, resembling malignant patterns during the active phase, while aligning more closely with benign patterns under passive conditions. This duality reflects partial angiogenic activation that is insufficient to generate a robust thermal signature at rest, a phenomenon consistent with the progressive stages of carcinogenesis described in angiogenesis literature [26,27,28]. Indeed, seminal work by Folkman and colleagues established that tumors cannot grow beyond 1–2 mm^3^ without inducing angiogenesis [26,27], providing a biological explanation for why thermal signatures intensify only after vascular expansion.

The high specificity of passive thermography (100%) observed in this study is comparable to findings from previous investigations, suggesting that passive imaging may be better suited for confirming benignity than detecting malignancy [10,12]. Conversely, the modest specificity of active thermography (75%) resulted from a small number of benign lesions that exhibited elevated Δ*T* responses. Similar findings have been reported in other thermographic studies and may reflect inflammatory processes, reactive vascular changes, or anatomical variability in cooling rather than true malignancy [11,12].

The ROC-derived cutoff values should be interpreted as exploratory and data-driven, requiring external validation before clinical implementation. In the present study, the Δ*T* ≥ 0.5 °C threshold was prespecified and applied for the primary binary classification based on established thermographic principles. In contrast, the 0.70 °C cutoff identified for active thermography was derived post hoc from ROC analysis using the Youden index. This distinction is important, as the prespecified threshold reflects a theoretically grounded reference value, whereas the ROC-derived cutoff represents statistical optimization within this specific cohort. Nevertheless, ROC analysis reinforced the superiority of active over passive thermography. Active Δ*T* measurements yielded an area under the curve (AUC) of 0.871, indicating excellent discriminative ability and strong separation between malignant and benign lesion profiles following dynamic cooling. The optimal cutoff of 0.70 °C provided high sensitivity (0.911) and moderate specificity (0.750), supporting the concept that thermal recovery kinetics capture physiologically meaningful differences in perfusion and metabolic activity. In contrast, passive thermography produced a lower AUC of 0.650, with its optimal cutoff (−0.20 °C) yielding reduced specificity (0.500) despite moderate sensitivity (0.857). These findings indicate that resting thermal differences alone are insufficient for robust lesion discrimination and highlight the added diagnostic value of dynamic thermographic stress.

From a translational perspective, recent systematic reviews evaluating infrared thermography combined with artificial intelligence have demonstrated that machine learning classifiers consistently outperform manual or threshold-based approaches across multiple oncologic applications [19,29]. Furthermore, AI-enhanced thermographic frameworks have shown promising predictive performance in treatment-related toxicity and malignancy detection in head-and-neck and breast cancer populations [18,30]. These data suggest that integrating active thermographic acquisition with automated feature extraction or deep learning may further enhance diagnostic robustness beyond the performance observed in the present study.

The study population represents a pre-selected cohort from a tertiary cancer center, resulting in an imbalanced distribution of malignant and benign lesions (56 vs. 12 cases), which may limit the generalizability of these findings to population-based screening settings. This distribution reflects the referral nature of the institution, where the prevalence of malignancy is inherently higher than in screening populations. In such imbalanced datasets, overall accuracy and predictive values may be influenced by disease prevalence. For this reason, sensitivity and specificity were prioritized as class-conditional measures, and ROC analysis was used as a prevalence-independent assessment of discrimination. Nevertheless, the relatively small number of benign lesions may have reduced the precision of specificity estimates, as reflected by wider confidence intervals. Additional limitations should also be acknowledged. As reported in previous studies [12,14,15], achieving uniform and consistent cooling across anatomical regions was technically challenging and may have contributed to Δ*T* variability. Furthermore, the absence of detailed pathological parameters, such as Breslow thickness, Clark level, ulceration status, or peritumoral inflammation, restricted more granular analysis of thermographic behavior. Studies incorporating such parameters have demonstrated meaningful correlations between thermal features and biological aggressiveness [11,13,15], suggesting that future investigations could strengthen diagnostic interpretation by integrating detailed histopathological data. The imbalance between lesion subtypes also limited comparative analyses, reflecting a common limitation in thermography studies, where SCCs and melanomas are often underrepresented relative to BCCs [4,11]. Finally, the designation of “benign lesions” encompasses a heterogeneous group. Inflammatory benign lesions, in particular, may exhibit thermographic patterns overlapping with malignancy, an aspect not systematically evaluated in the present study and deserving further investigation. Because this was a single-center diagnostic accuracy study conducted under standardized acquisition conditions, external validation in independent cohorts and multicenter settings will be necessary before broader clinical implementation.

Recent dermatologic reviews have similarly emphasized the need for standardized acquisition protocols, multicenter validation cohorts, and integration with dermoscopic or multimodal imaging pipelines before thermography can be widely implemented in routine dermatologic oncology practice [31]. These recommendations align closely with the methodological refinements suggested by our findings.

Overall, these results reinforce the potential of active infrared thermography as a non-invasive and low-cost adjunct for skin cancer triage. The technique may be particularly relevant in resource-limited settings, mobile screening initiatives, or population-based programs where rapid physiological assessment can complement visual inspection. Nevertheless, infrared thermography should not be considered a standalone diagnostic method. In parallel with functional imaging approaches such as thermography, structural optical imaging modalities—particularly optical coherence tomography (OCT) and OCT angiography (OCTA)—have demonstrated the capacity to provide high-resolution microvascular and morphological characterization of human skin in vivo. Recent advances enabling wide-field, spectrally extended line-field OCTA have expanded the field of view while preserving capillary-level resolution in cutaneous imaging, supporting its applicability to dermatologic assessment. In addition, developments in artifact-free and deblurred OCT reconstruction frameworks have improved image clarity and structural delineation, further enhancing the reliability of optical structural imaging. These advances suggest that thermographic functional assessment and structural optical imaging may represent complementary approaches within multimodal diagnostic pipelines rather than competing modalities [32,33].

Future studies should focus on larger and more diverse populations, standardized acquisition protocols, and integration with dermoscopic assessment or computational tools to refine diagnostic thresholds and enhance predictive accuracy. As quantitative approaches evolve, including deep learning applied to dynamic thermographic sequences [16] and physiologically informed thermal modeling [17], infrared imaging may increasingly contribute to multistep diagnostic pathways for cutaneous malignancies.

## 5. Conclusions

In this diagnostic accuracy study, active infrared thermography demonstrated superior performance compared with passive thermography for differentiating malignant from benign skin lesions. Cooling-induced thermal recovery significantly enhanced temperature differentials, resulting in higher sensitivity, improved overall accuracy, and a favorable ROC profile, whereas passive thermography showed limited sensitivity despite high specificity. These findings indicate that dynamic thermal stimulation is essential for revealing physiologically relevant vascular and metabolic alterations associated with cutaneous malignancies.

Although infrared thermography should not replace established diagnostic methods, active thermography may represent a useful adjunctive tool for skin cancer assessment, particularly in triage or screening-oriented settings where non-invasive and low-cost approaches are desirable. Further studies with larger cohorts, standardized acquisition protocols, and integration with clinical, dermoscopic, or computational strategies are warranted to better define its role within multimodal diagnostic pathways for early skin cancer detection.

## Figures and Tables

**Table 1:** Clinical, Demographic, and Lesion Characteristics of the Study Sample.

Category	Variable	*n* (%)
Patient characteristics	Number of patients	64 (100%)
	Age	
	18–30	0 (0%)
	31–40	5 (7.8%)
	41–50	5 (7.8%)
	51–60	16 (25%)
	>60	38 (59.4%)
	Sex	
	Male	30 (46.9%)
	Female	34 (53.1%)
Lesion characteristics	Total lesions evaluated	100
	Lesions included in accuracy analysis	68
	Number of lesions per patient, *n* (%)	
	1	
	2	49 (75%)
	3	5 (7.8%)
	4	4 (6.25%)
	5	4 (6.25%)
		3 (4.7%)
	Lesion location	
	Head/neck	48 (48%)
	Trunk	24 (24%)
	Upper limbs	18 (18%)
	Lower limbs	10 (%)
	Clinical diagnostic hypothesis	51 (51%)
	BCC	22 (22%)
	SCC	9 (9%)
	Melanoma	6 (6%)
	Premalignant	5 (5%)
	Benign	7 (7%)
	Missing	
	Concordance of clinical diagnosis with histopathology	85.55%
Histopathology		Included in accuracy analysis/Total
	Malignant lesions	56/68 (82.3%)
	BCC	38/48 (79.2%)
	SCC	16/18 (88.9%)
	Melanoma	2/2 (100%)
	Benign lesions	12/23 (52.2%)
	Premalignant lesions	0/9 (0%)
Thermography acquisition	Excluded lesions due to inadequate cooling	24 (24%)
	Δ*T* (active), mean ± SD	4.57 ± 4.3
	Δ*T* (passive), mean ± SD	0 ± 0.6
	Acclimatization time	10 min
	Room temperature	24 °C

**Table 2:** The 2 × 2 contingency tables for Active and Passive Infrared Thermography compared with histopathology.

Technique	Malignant (Reference Standard)	Benign (Reference Standard)	Total
A. Active Thermography			
Test Positive	TP = 51	FP = 3	54
Test Negative	FN = 5	TN = 9	14
Total	56	12	68
B. Passive Thermography			
Test Positive	TP = 10	FP = 0	10
Test Negative	FN = 46	TN = 12	58
Total	56	12	68

**Table 3:** Diagnostic accuracy metrics for Active versus Passive Infrared Thermography.

Metric	Active Thermography		Passive Thermography		*p*-Value *
	% (95% CI)	*n*/N	% (95% CI)	*n*/N	
Sensitivity	91.1% (80.4–97.0)	51/56	17.9% (8.9–30.4)	10/56	<0.0001
Specificity	75.0% (42.8–94.5)	9/12	100% (73.5–100)	12/12	0.25
Accuracy	88.2% (78.1–94.8)	60/68	32.4% (21.5–44.8)	22/68	- ^1^

* *p*-Values calculated using McNemar’s test for paired proportions (malignant and benign subsets separately). ^1^ Accuracy was not formally compared using a paired statistical test, as McNemar evaluates sensitivity and specificity components rather than overall accuracy.

## Data Availability

The data presented in this study are available on request from the corresponding author. The data are not publicly available due to ethical and privacy restrictions related to patient information.

## Supplementary Materials

The following supporting information can be downloaded at https://www.mdpi.com/article/10.3390/cancers18050829/s1. Table S1: Mean temperature differentials (Δ*T*) between lesions and adjacent healthy skin. Table S2: Paired comparison of diagnostic performance between active and passive infrared thermography using McNemar’s test.

## Abbreviations

AUC	Area Under the Curve
AT	Active Thermography
BCC	Basal Cell Carcinoma
CI	Confidence Interval
Δ*T*	Temperature Differential between Lesion and Adjacent Healthy Skin
FN	False Negative
FP	False Positive
IR	Infrared
IT	Infrared Thermography
LWIR	Long-Wave Infrared
PT	Passive Thermography
ROC	Receiver Operating Characteristic
SCC	Squamous Cell Carcinoma
TN	True Negative
TP	True Positive
VEGF	Vascular Endothelial Growth Factor
