# Supplementary material for: Active Versus Passive Infrared Thermography for Skin Cancer Detection: A Diagnostic Accuracy Study
Source: Cancers (Basel). 2026 Mar 4;18(5):829. doi: 10.3390/cancers18050829 (PMC12985089; doi:10.3390/cancers18050829)
Supplement: Supplementary file 1 [file cancers-18-00829-s001.zip › cancers-4155854-supplementary.pdf]

SUPPLEMENTARY MATERIAL

Supplementary Table S1. Mean Temperature Differential (ΔT) Between Lesions and Adjacent Healthy Skin

Type of Lesion	Active Thermography (AT)ΔT (°C), mean ± SD	Passive Thermography (PT)ΔT (°C), mean ± SD
Malignant	5.38 ± 4.51	0.10 ± 0.50
Pre-malignant	4.69 ± 2.59	–0.55 ± 1.15
Benign	0.69 ± 1.54	–0.14 ± 0.35

Paired statistical comparison of diagnostic performance

A paired comparison was performed using McNemar’s test to assess whether the two thermographic approaches differed significantly in their ability to correctly classify lesions.

Sensitivity comparison (malignant lesions only, n = 56)

Active thermography correctly classified 41 malignant lesions that were missed by passive thermography, while no malignant lesion was correctly classified by the passive technique and misclassified by the active technique. McNemar’s test confirmed a significant difference in sensitivity between the two methods ($p < 0.0001$).

Specificity comparison (benign lesions only, n = 12)

Passive thermography correctly classified all benign lesions, whereas active thermography produced three false positives. Despite this numerical difference, McNemar’s test showed no statistically significant difference in specificity ($p = 0.25$), likely due to the limited number of benign cases.

Supplementary Table S2. Paired comparison of diagnostic performance between Active and Passive Infrared Thermography using McNemar’s test

Comparison	Category	Active Correct / Passive Incorrect	Passive Correct / Active Incorrect	p-value (McNemar)
Sensitivity	Malignant lesions (n = 56)	41	0	**< 0.0001**
Specificity	Benign lesions (n = 12)	0	3	0.25
